# MIGAN: Mutual-Interaction Graph Attention Network for Collaborative Filtering

**DOI:** 10.3390/e24081084

**Published:** 2022-08-05

**Authors:** Ahlem Drif, Hocine Cherifi

**Affiliations:** 1 Faculty of Sciences, Ferhat Abbas University, Setif 1, Setif 19000, Algeria; 2 Laboratoire d’Informatique de Bourgogne, University of Burgundy, 21078 Dijon, France

**Keywords:** recommender systems, mutual influence, graph attention network, self-supervised, collaborative filtering

## Abstract

Many web platforms now include recommender systems. Network representation learning has been a successful approach for building these efficient recommender systems. However, learning the mutual influence of nodes in the network is challenging. Indeed, it carries collaborative signals accounting for complex user-item interactions on user decisions. For this purpose, in this paper, we develop a Mutual Interaction Graph Attention Network “MIGAN”, a new algorithm based on self-supervised representation learning on a large-scale bipartite graph (BGNN). Experimental investigation with real-world data demonstrates that MIGAN compares favorably with the baselines in terms of prediction accuracy and recommendation efficiency.

## 1. Introduction

In the literature, there are numerous techniques for building recommendation systems. They can be classified either as collaborative, content-based, or hybrid filtering approaches [1,2,3,4]. Collaborative filtering is the most influential. It relies on identifying users with similar tastes for item recommendations. It leverages their feedback to make suggestions to the active user. Collaborative recommender systems have been implemented in multiple application areas [5,6,7].

Learning effective user/item representations from their interactions and side information in recommender systems is a challenging issue. Since most data have a graph structure and the graph neural network (GNN) has superiority in representation learning, using GNN in recommender systems is a flourishing field of research. Several works are based on GNN to perform recommendations, and other tasks, such as the graph convolutional network (GCN) [8] and graph attention network (GAT) [9]. GAT computes the representation of nodes by adaptively combining their neighborhoods’ vectors using a self-attention mechanism with trainable attention weights. Wang et al. [10] suggest a knowledge graph attention network (KGAT) for KG-based recommendations. To enrich the representation, the authors consider the implicit collaborative information of multi-hop neighbors. In the work [11], the authors propose a neighbor-aware graph attention network for recommendation tasks to model the implicit correlations of neighbors. Unlike previous attention networks, our current work determines the most relevant weights characterizing the mutual influence among item-users. In addition to learning the interaction between user interests and item embeddings, it integrates a new component accounting for the mutual influence of items carrying collaborative signals on user decisions. MIGAN learns deeply the most relevant weights representing the users mutual influence on an item. It exploits the complex relation between the user profile and the item attributes. Its main advantage lies in its ability to discriminate the relative influence of various interactions between nodes. Our main contributions summarize as follows:

- Our approach is based on self-supervised representation learning on a large-scale bipartite graph (BGNN). We have adapted this powerful representation for the recommendation task because its ability to model the dependencies between the nodes on a large scale.
- The collaborative filtering recommender based on interactive neural attention networks takes advantage of the encoding potential of interactive attention between users and items. It learns the most significant weights representing users’ mutual effect on the item. Consequently, exploiting this information improves the recommender systems’ accuracy.
- The empirical evaluation, including various real-world dataset, shows that MIGAN significantly outperforms the state-of-the-art baselines.

The rest of the paper is organized as follows. Section 2 describes the related literature. Section 3 presents in detail the proposed architecture. Section 4 discusses the experimental results. Section 5 summarizes the conclusions.

## 2. Related Work

Graph embedding models are one of machine learning’s newest and fastest-growing subfields. Its strength is in its ability to take advantage of the intrinsic graph structure of a wide range of data types encountered in a wide range of applications. The graph format models a set of elements (represented by nodes) and their relationships (represented by edge) to capture structural information. Therefore, there have been proposed various graph embedding models in literature [12,13]. Node2Vec is an embedding model for converting graphs into numerical representations where each node in the network is used as a starting point to produce a corpus of random walks [14]. In a first-order random walk, each step is solely determined by the current state. The steps in a second-order random walk are determined by the current and prior states. The random walk corpus is fed through Word2Vec to build the node embeddings. Liang et al. [15] extend the variational autoencoders (VAEs) to collaborative filtering for implicit feedback. It models the collaborative information into multinomial likelihood (MultiVAE) for the data distribution to sample prediction for items on the long tail. Drif et al. [16] develop an ensemble variational autoencoder framework for recommendations (EnsVAE) that specifies a procedure to transform sub-recommenders’ predicted utility matrix into interest probabilities that allow the VAE to represent the variation in their aggregation. This architecture is based on two components: (1) GloVe content-based filtering recommender (GloVe-CBF) that exploits the strengths of embedding-based representations and stacking ensemble learning techniques to extract features from the item-based side information, and (2) a variant of neural collaborative filtering recommender, named the Gate Recurrent Unit-based Matrix Factorization (GRU-MF) recommender. It models a high level of non-linearities and exhibits interactions between users and items in latent embeddings, reducing user biases towards items that are rated frequently by users. Weng Lo et al. [17] suggested the graph flow data’s extensive structural information based on a graph neural networks (GNNs). The latter works with the message passing concept, where a node collects its neighbors features and sends them to a node as a message. In recent years, the graph attention network approach has developed rapidly. Wang et al. [18] introduced a multi-dimension interaction-based attentional knowledge graph neural network (MI-KGNN) to improve recommendations based on knowledge graph (KG). MI-KGNN explores the interaction between users and the neighborhood during embedding propagation. In [10], the authors proposed a knowledge graph attention network (KGAT) modeling the high-order connectivity in knowledge graphs. It exploits the attention mechanism to determine important neighbors. In [19], the authors propose a multi-view graph attention network (MV-GAN) based on the heterogeneous information networks for the recommendation. They create attention networks at the node and path levels to learn user and product representations from every view. A view-level attention mechanism is developed to integrate various relationship types in multiple views co-operatively. Liu et al. [20] propose a contextualized graph attention network (CGAT) based on an entity’s local and non-local context data in a knowledge graph. CGAT implements a graph attention method to record local context information while considering users’ unique preferences for entities. The non-local context of an entity is also extracted using a biased random walk sampling method by CGAT. In fact, propagating information from nodes across the network is done during numerous iterations. The aggregated information at each node (node embedding) is a memory- and time-consuming task. Due to this fact, many GNNs for recommendation suffer from scalability limitations, unpredictable memory, and computational resource requirements on large graphs. To overcome these drawbacks, our work considers node representation learning on large-scale bipartite graphs.

## 3. Mutual-Interaction Graph Attention Network Approach

**Figure 1:**
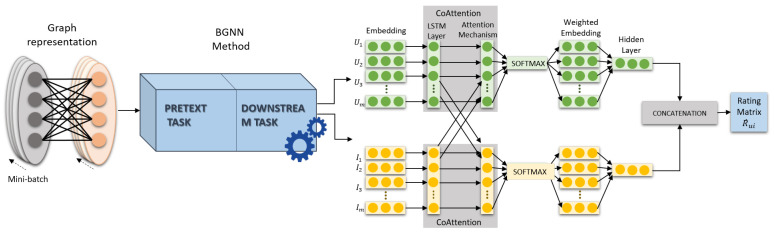
MIGAN Architecture.

The proposed architecture considers the mutual collaborative information of the user’s preference on the whole neighboring item to enrich the representation. It is based on self-supervised representation learning on a large-scale bipartite graph (BGNN) [21]. Figure 1 illustrates the model architecture in detail. We first formulate the recommendation task on the bipartite graph. Secondly, we introduce the embedding representation based on BGNN and then describe the mutual interaction graph attention mechanism.

### 3.1. Problem Formulation

Let $U=u_{1},u_{2},\ldots ,u_{n}$ and $I=i_{1},i_{2},\ldots ,i_{m}$ be the sets of users and items, respectively, where *n* is the number of users, and *m* is the number of items. We assume that $R^{n\times m}$ is the user-item rating matrix.

We formulate the recommendation task as a prediction problem as follows:

$\hat{R}$: utility matrix; ${\hat{r}}_{u}$: predicted rating for each user u∈U; $U=u_{1},u_{2},\ldots ,u_{n}$: is the sets of users, where *n* is the number of users; $I=i_{1},i_{2},\ldots ,i_{m}$ is the set of the items, where *m* is the number of items; $R_{ui}$: is the ground truth rating assigned by the user *u* on the item *i*.

We define the utility matrix as:

(1) $${\hat{R}}_{ui}=PQ^{T}=\sum_{k=1}^{K}p_{uk}q_{ki}$$

where: *K* is latent space’s dimension.

$S_{\left(n\times m\right)}^{\prime }$: is the inner product of both user and item latent vectors. It is decomposed by the matrix factorization method into $P\in R^{N\times K}$ and $Q\in R^{M\times K}$.

To normalize $\hat{R}$, we apply the min/max scaling:

(2) $$minmax\left(x\right)=\frac{x-min}{max-min}\;\forall x\in \hat{r}\left(ui\right)$$

where: $min=\min\left(r_{ui}\right)$ is the minimal rating;

$max=\max\left(r_{ui}\right)$: is the maximal rating. The normalization eliminates user bias. In other words, users have different ways to rate the items. Some would only give high ratings to items they like, while others do the opposite. Normalizing users’ ratings hide their bias by mapping them to values between 0 and 1. The lowest rating of each user is associated with 0, while 1 represents their highest value. It assists in leveraging more accurate collaborative-based recommendations.

Table 1 reports the notations used in the rest of this paper.

A bipartite graph is composed of two independent sets of vertices, $U_{1}$ and $I_{1}$. The edges connect a vertex from one set $U_{1}$ to one in $I_{1}$.

We define Bipartite Graphs as follows: Let $G=(U_{1},I_{1},E)$ be a bipartite graph. $e_{ij}$ represents the edge between $u_{i}$ and $i_{j}$.

$B_{u}\in R^{M\times N}$ is the incidence matrix for $U_{1}$. $B_{i}\in R^{N\times M}$ is the incidence matrix for $I_{1}$. Where

(3) $$B_{u}\left(i,j\right)=\left\{\begin{array}{cc} 1 & \mathrm{if}\;e_{ij}\in E,\\ 0 & \mathrm{if}\;e_{ij}\notin E. \end{array}\right.$$

$X_{u}\in R^{M\times P}$: is defined as a feature matrix of node $u_{i}$ ($X_{i}$ is similarly written).

Our work is based on the self-supervised node representation learning model [21] that can employ topology information as well as separate node attributes from two domains to increase the recommendation performance for large graph.

### 3.2. Embedding Representation Based on Bipartite Graph Neural Networks (BGNN)

He et al. [21] proposed a a self-supervised representation learning framework for large-scale bipartite graphs. In this section, we adapt the outputs of this BGNN architecture, thus, we can deploy it in our recommendation system. Let us define the following notions: $H_{u}\in R^{P\prime }$ ($H_{i}\in R^{Q\prime }$, respectively): is nodes embedding representation for $U_{1}$ ($I_{1}$, respectively). $f_{emb}$: is an embedding model given by θ parameters.

The embedding of distinct node features $X_{u}$ and $X_{i}$ is written as:

(4) $$H_{u},H_{i}=f_{emb}\left(X_{u},B_{u},X_{i},B_{i},\theta \right)$$

The architecture of $f_{emb}$ is based on two functions: (i) Inter-Domain Message Passing (IDMP) and (ii) Intra-Domain Alignment (IDA). We will describe briefly these functions and show how we prepare the outputs for the recommendation task (interested reader can refer to [21] for more details). IDMP enables one domain to aggregate information from the other domain, through the linked edges, as follows:

(5) $$H_{i\to u}=f_{u}\left(X_{i},B_{u},\theta \right)$$

(6) $$H_{u\to i}=f_{i}\left(X_{u},B_{i},\theta \right)$$

such as: $f_{u}$ (resp. $f_{i}$): represents the IDMP function for this domain.

$H_{i\to u}$ (resp. $H_{u\to i}$): is the flow of aggregated information from $I_{1}$ (resp. $U_{1}$) to $U_{1}$ (resp. $I_{1}$). After that, the Intra-Domain Alignment (IDA) is deployed for theses two distinct features into a single representation. After the self-supervised training, the algorithm gives the domains representation of $H_{u}^{1}$ and $H_{I}^{1}$. The adversarial loss $L_{adv}$ is used to compute the best results.

(7) $$Loss_{u}=L_{adv}\left(H_{i\to u},X_{u}\right)$$

(8) $$Loss_{i}=L_{adv}\left(H_{u\to i},X_{i}\right)$$

Thus, the Inter-Domain Message Passing (IDMP) is expressed as:

(9) $$H_{i\to u}^{\left(K\right)}=\sigma \left(B_{u}^{{}^{\prime }}H_{i}^{\left(K\right)}W_{u}^{K}\right)$$

(10) $$H_{u\to i}^{\left(K\right)}=\sigma \left(B_{i}^{{}^{\prime }}H_{u}^{\left(K\right)}W_{i}^{K}\right)$$

where: $B_{u}^{{}^{\prime }}=D_{u}^{-1}B_{u}$ is the normalization of $B_{u}$ ($D_{u}$ is the degree matrix of $B_{u}$). By normalizing the incidence matrix of the graph, the algorithm can effectively reduce the computational cost. σ is the activation function ReLU [22]. *K* denotes the depth index of the hidden features of the nodes in set $U_{1}$ (resp. $I_{1}$).

Let σ is the Intra-Domain Alignment (IDA) discriminator and ϕ is the IDMP generator. The discriminator loss function is expressed as follows:

(11) $$L_{D}\left(\sigma |\phi \right)=\frac{1}{M}\sum_{j=1}^{M}logP_{\sigma ,\phi }\left(source=0|h_{u(j)}\right)-\frac{1}{N}\sum_{j=1}^{N}logP_{\sigma ,\phi }\left(source=1|h_{i\to u(j)}\right)$$

where: $P_{\sigma ,\phi }$(source=1|h) is the probability that the input feature vector *h* is from the source domain $H_{i\to u}$.

The implementation for the self-supervised representation learning on large scale bipartite graph (BGNN) for the recommendation task is summarized in Algorithm 1.
**Algorithm 1** Bipartite Graph Neural Networks for recommendation task.**Input** $X_{u}:User\;features\;list$ $X_{i}:Item\;features\;list$ R:Ratingmatrix **Output** $\;\;\;\;\;\;\;emb_{u}:Users^{\prime }\;Embedding\;representation\;based\;on\;BGNN.$ $\;\;\;\;\;\;\;emb_{i}:Items^{\prime }\;Embedding\;representation\;based\;on\;BGNN.$ **Begin**   **phase 1**                               ▹ Extract the graph from the rating matrix $\;\;\;\;\;\;\;\;\;\;B_{u},B_{i}=GetBipartiteGraph\left(R\right)$   **phase 2**                     ▹ Computing embeddings for each item and user $\;\;\;\;\;\;\;\;\;\;H_{u}^{0}=X_{u}$
$\;\;\;\;\;\;\;\;\;\;\;\;H_{i}^{0}=X_{i}$            **for**
j=0,1,…,K
**do** $\;\;\;\;\;\;\;\;\;\;\;\;\;\;\;H_{u\to i}^{j}=\mathrm{IDMP}(B_{i},H_{u}^{K})$ $\;\;\;\;\;\;\;\;\;\;\;\;\;\;\;H_{i\to u}^{j}=\mathrm{IDMP}(B_{u},H_{i}^{K})$ $\;\;\;\;\;\;\;\;\;\;\;\;\;\;\;H_{u}^{K}=\mathrm{IDA}(H_{u\to i}^{j})$ $\;\;\;\;\;\;\;\;\;\;\;\;\;\;\;H_{i}^{K}=\mathrm{IDA}(H_{i\to u}^{j})$   **   Endfor**   **phase 3**                                                         ▹ embeddings preparation $\;\;\;\;\;\;\;\;\;\;emb_{u}=GetEmbeddings\left(H_{u}^{K}\right)$ $\;\;\;\;\;\;\;\;\;\;emb_{i}=GetEmbeddings\left(H_{i}^{K}\right)$ $\mathrm{return}\;\;\;\;\;\;emb_{u},emb_{i}$

### 3.3. The Interactive Attention Network Recommender

**Figure 2:**
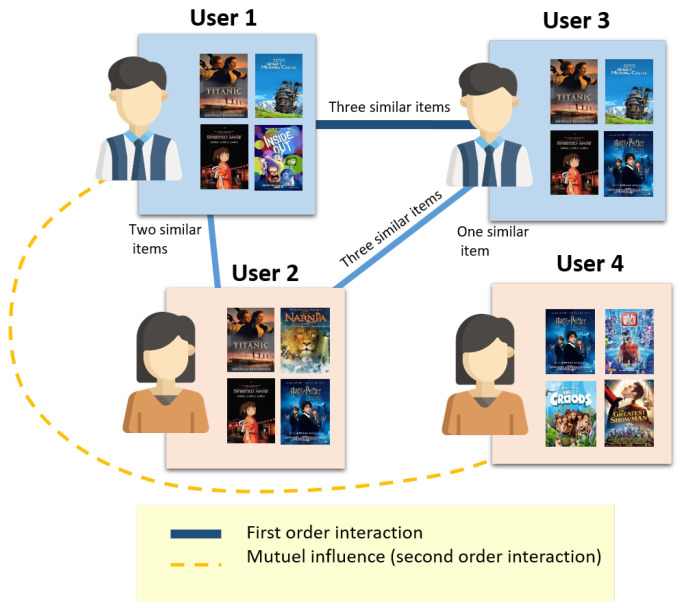
The interactive attention network recommender. In this example, user 1 and user 2 rate three similar items. They have strong interactive attention. User 2 and user 3 rate an item not seen by user 1. Therefore, one can deduce that this new item can also attract user 1. It is a first-order interaction. Moreover, one can deduce a mutual influence based on entities’ dependencies at more than a first order interaction level. For example, user 1 influences user 4 (a similar user of user 3), generating a recommendation based on user 3 preferences.

The interactive attention network recommendation system aim at identifying latent features that show the users and items mutual influence. The attention mechanism has been shown to be useful in a variety of machine learning applications, including image/video captioning [23,24]. Our proposed interactive concept extracts each participant’s contribution from their compressed representation. Thus, it allows the proposed recommendation framework to model efficiently the interaction characteristic. This attention network model figures out which weights best represent the users’ mutual effect on the item. Figure 2 explains the mutual interactions between users and items.

Algorithm 2 shows the architecture of the proposed interactive attention network. In order to anticipate a distribution over the items, we create combined user and item interactive attention maps. As a result, the co-attention mechanism detects a correlation between items and users and calculates the likelihood that an item will be of interest to indirect comparable individuals.

The first embedding layers $e_{u}$ and $e_{i}$ captures latent features of users $p_{u}$ and items $q_{i}$. They are followed by Long-Short-Term-Memory (LSTM) layers to learn long sequences with long time lags. Each LSTM state includes two inputs: the current feature vector and the output vector $h_{t-1}$ from the previous state. Its output vector is $h_{t}$. We chose to apply the LSTM model as it exhibits interactions between users and items in latent embeddings. Each node embedding layer is chained with an LSTM layer that contains recurrent modules enabling long-range learning. Information from nodes neighbors gradually enhances the subsequent feature representation because LSTM has an augmented hidden state with non-linear mechanisms. It allows propagating without modification, updating, or resetting states using simple learned gating functions. The LSTM representation is expressed as follows:

(12) $$h_{tu}=g_{1}\left(p\right)$$

(13) $$h_{ti}=g_{2}\left(q\right)$$

The learned representation is $H_{p}$ and $H_{q}$, respectively, with d×n dimensions for $H_{p}$ and d×m for $H_{q}$. Users and items embedded inputs are projected into a vector representation space using the attention technique. In fact, this representation models the high-order non-linear mutual relationship. For the interactive attention mechanism, we build an attention maps in order to predict a distribution over the items. For this purpose, we compute a matrix $L=tanh(H_{p}^{⊤}W_{pq}H_{q})$, where $L\in R^{n\times m}$, and $W_{pq}$ is a d×d a learnable parameters matrix. The features co-attention maps is defined as:

(14) $$\begin{array}{c} \alpha_{p}^{*}=tanh\left(W_{p}H_{p}+\left(W_{q}H_{q}\right)L^{⊤}\right)\\ \alpha_{q}^{*}=tanh\left(W_{q}H_{q}+\left(W_{p}H_{p}\right)L\right) \end{array}$$

The interactive attention model uses a tangent function to model the mutual interactions between users and items. Afterward, we compute the the probability distribution over the embedding space. The softmax function is used to generate the attention weights:

(15) $$\alpha_{u}=Softmax\left(f\left(\alpha_{p}^{*}\right)\right)$$

(16) $$\alpha_{i}=Softmax\left(f\left(\alpha_{q}^{*}\right)\right)$$

where *f*: is a multi-layer neural network.

Then, the high order interaction latent space of users and items is given by:

(17) $$f_{1}=\left[\beta \prime_{u}⊕\beta \prime_{i}\right]$$

where $\beta_{p}$ and $\beta_{q}$: are the derived attention weights.

As a result, the predicted matrix $\hat{R_{ui}}$ is defined as:

(18) $$\hat{R_{ui}}=f\left(f_{1}\right)$$

where *f*: is a dense layer using a sigmoid activation function.

Finally, we train the model to minimize the loss function which is the Mean Absolute Error (MAE):

(19) $$L\left(R_{ui},{\hat{R}}_{ui}\right)=\frac{1}{\left|C\right|}\sum_{(u,i)\in C}\left|\left(R_{ui}-\hat{R_{ui}}\right)\right|$$

**Algorithm 2** CoAttention: The interactive attention network recommender.**Input**   lstmU: user’s lstm : size d×n   lstmI: item’s lstm: size d×m **Output** $\;\;\;\;\;{\mathrm{att}}_{ui}:The\;Interactive\;Attention\;between\;users\;and\;items$ **Begin**   **phase 1**                                                            ▹ initialization of weights      
$W_{u}:size\;n\times d$      
$W_{i}:size\;m\times d$      
$W_{ui}:size\;d\times d$      
$b_{u}:size\;d\times n$      
$b_{i}:size\;d\times m$   **phase 2**                                                          ▹ tanh function application      S = lstmI      G = lstmU      
$F=\tanh(S^{t}W_{ui}G)$      
$\alpha_{u}^{*}=\tanh(W_{u}G+\left(W_{i}S\right)F^{t})$      
$\alpha_{i}^{*}=\tanh(W_{i}S+\left(W_{u}G\right)F)$   **phase 3**                                                     ▹ Softmax function application      
$\alpha_{u}=\mathrm{softmax}(f\left(b_{u}\alpha_{u}^{*}\right))$ $\;\;\;\;\;\;\;\;\alpha_{i}=\mathrm{softmax}(f\left(b_{i}\alpha_{u}^{*}\right))$ $\;\;\;\;\;\;\;\;\beta_{u}=O\left(\alpha_{u}\right)$ $\;\;\;\;\;\;\;\;\beta_{i}=O\left(\alpha_{i}\right)$                     ▹ O: is the batch.dot() function from Keras backend that is used between two tensors (($\alpha_{p}{)}^{t}$ and ${\left(G\right)}^{t}$), ($\alpha_{(}{q)}^{t}$ and ${\left(S\right)}^{t}$) respectively.   **phase 4**            ▹ Each output of function *O* are transposed and then used as input into a product function. After that, both results ($\beta \prime_{u}$
$\beta \prime_{i}$) can be summed by a concatenate function.      
${\mathrm{att}}_{ui}=\mathrm{concatenation}\left(\beta \prime_{u},\beta \prime_{i}\right)$ return   
${\mathrm{att}}_{ui}$

Algorithm 3 summarizes the overall mutual-interaction graph attention network approach. Network representation learning can tackle the recommendation problems by embedding nodes into a low-dimensional space $R^{d}$. Furthermore, this adapted BGNN representation for recommendation task improves multi-hop relationship modeling and the training accuracy. Unlike previous research on graph neural networks for recommendation that only learn complex relationship between the target and their neighbors using attention network, our work learn the most important weights representing the users’ mutual influence on the item based on the interactive attention.
**Algorithm 3** Migan: Mutual-Interaction Graph Attention Network.**Input** $\;\;\;\;\;\;\;X_{u}:User\;features\;list$ $\;\;\;\;\;\;\;X_{i}:Item\;features\;list$ U:Listofuser:size=n I:Listofitem:size=m R:Ratingmatrix $\mathrm{Output}\;P_{ui}:prediction\;matrix$ **Begin**   **phase 1**                                    ▹ Preparing data to be passed to the BGNN
foreachu∈U,i∈VdoRj=minmax(R)   **phase 2**                                       ▹ extracting embeddings by BGNN-Class()             $\;\;\;\;\;\;\;\;\;\;\;\;\;emb_{u},emb_{i}=BGNN\left(X_{u},X_{i},R_{j}\right)$   **phase 3**              ▹ User and Item embedding are followed by LSTM layers.
$\;\;\;\;\;\;\;\;\;\;\;\;\;\;\;\;\;\;\;\;\;\;\;\;\;\;\;\;\;\;\;\;\;\;\;\;lstm_{u}=LSTM\left(emb_{u}\right)$ $\;\;\;\;\;\;\;\;\;\;\;\;\;\;\;\;\;\;\;\;\;\;\;\;\;\;\;\;\;\;\;\;\;\;\;\;lstm_{i}=LSTM\left(emb_{i}\right)$   **phase 4**                                                      ▹ Applying Attention mechanism
$\;\;\;\;\;\;\;\;\;\;\;\;\;\;\;\;\;\;\;\;\;\;\;\;\;\;\;\;\;\;\;\;\;\;\;\;att_{u}=Attention\left(lstm_{u}\right)$ $\;\;\;\;\;\;\;\;\;\;\;\;\;\;\;\;\;\;\;\;\;\;\;\;\;\;\;\;\;\;\;\;\;\;\;\;att_{i}=Attention\left(lstm_{i}\right)$ $\;\;\;\;\;\;\;\;\;\;\;\;\;\;\;\;\;\;\;\;\;\;\;\;\;\;\;\;\;\;\;\;\;\;\;\;att_{ui}=CoAttention\left(lstm_{u},lstm_{i}\right)$   **phase 5**                                                            ▹ Concatenating The outputs
$\;\;\;\;\;\;\;\;\;\;\;\;\;\;\;\;\;\;\;\;\;\;\;\;\;\;\;\;\;\;\;\;\;\;\;\;ATT=concatenation(att_{u},att_{i},att_{ui})$                                     InteractiveAttention = BuildModel(ATT);                                     InteractiveAttention.trainModel(D);
$\mathrm{break}\mathrm{return}\;\;\;\;P_{ui}=InteractiveAttention.predict\left(\varphi \right)$

## 4. Experiments and Discussion

We conduct our experiments on MovieLens 1M that is commonly used for benchmarking recommendation frameworks. The MovieLens dataset [25] is a real, timestamped 5-star ratings of the MovieLens platform users on various films. The selected dataset contains 1 million ratings from 6000 users over 4000 movies. Table 2 shows the dataset description. We divide the datasets into 75% training data and 25% testing data in a stratified way.

We evaluate MIGAN using Mean Average Precision and Normalized Discounted Cumulative Gain. The mean average precision (MAP) measures the accuracy of information retrieval. The results of recommender systems are frequently pruned to return the Top-k components. The value of k varies based on the application: a system might display the top three trending items or the best ten items that meet the current user’s preferences. The Normalized Discounted Cumulative Gain (NDCG) calculates an item’s normalized usefulness depending on its position in the final list. It is used to assess the Top-k recommended items’ ranking quality. Interested readers can refer to [16] for more details.

### 4.1. Hyperparameters Analysis

Here, we report the hyperparameters analysis phase, which is performed separately for each MIGAN recommender variant. Evaluation is done with MAP@k and NDCG@k.

The main idea of our model is to pass the results of the BGNN through a neural network and then apply the attention model to it. Consequently, we perform the analysis on 1–6 variants and focus on the learning algorithms used by each hyperparameter. We have 3 hyperparameters and two kinds of recurrent networks: Embedding output size = 50, 75, 100, Neural network = LSTM, GRU. It gives us six variants. The architecture of each variant is as follows:

- **variant 1:** BGNN output size =75×1 | Neural Network = LSTM.
- **variant 2:** BGNN output size = 100×1 | Neural Network = LSTM.
- **variant 3:** BGNN output size = 50×1 | Neural Network = LSTM.
- **variant 4:** BGNN output size = 75×1| Neural Network = GRU.
- **variant 5:** BGNN output size = 100×1| Neural Network = GRU.
- **variant 6:** BGNN output size = 50×1 | Neural Network = GRU.

As shown in Table 3, **Variant 1** scores better than the other variants. Thus, it is picked for further tweaking.

**Figure 3:**
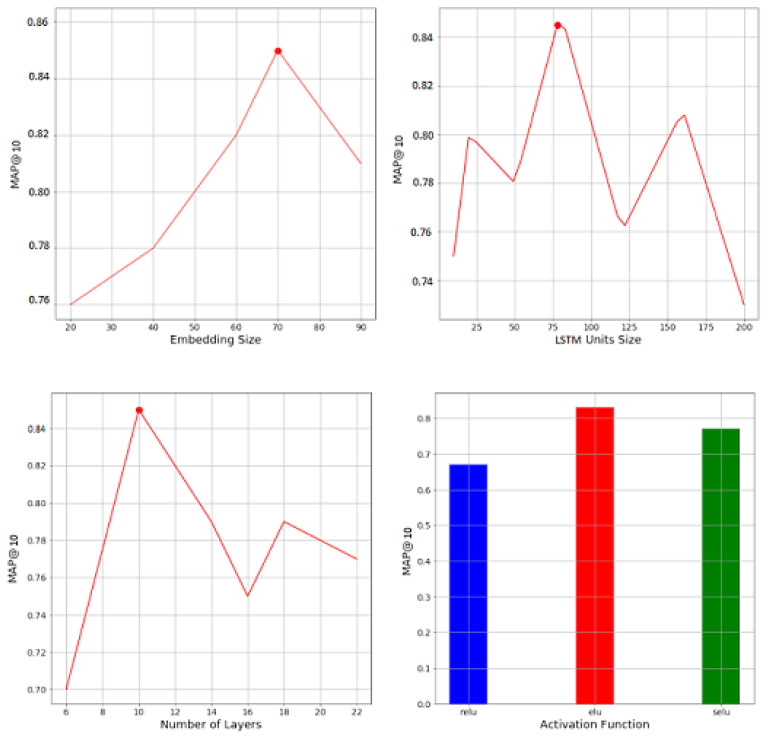
Hyperparameter searching MIGAN filtering recommendation system.

We generate the utility matrix based on the learned embeddings. Figure 3 illustrates the hyperparameters analysis.

We explore a range of values for each hyperparameter as reported below:

- Dimensions of the embedding α∈[30,100];
- Number of dense layers after the co-attention θ∈[2,20];
- Number of neurons per dense layer τ∈[30,150];
- Activation function used in the dense layers σ∈{selu,elu,relu};
- Optimizer λ∈{sgd,adam,adagrad}.

Results illustrate that we achieve the best performance for the following settings: α=50, θ=3, τ=100, σ=elu, λ=Adam.

### 4.2. Performance Comparison with the Baselines

In this subsection, MIGAN’s final benchmark results are compared to the outcomes of certain baseline recommender systems. We execute the baselines in the same evaluation environment as MIGAN to guarantee that comparisons are fair. Furthermore, we deploy the *NeuRec* library. Furthermore, we deploy the *NeuRec* library. It is released on GitHub as open-source software under an MIT license, and it implements 33 neural recommender systems [26].

We compare the proposed recommender framework with the following baselines:

- *The stacked content-based filtering recommender:* the work [16] developed a content-based recommender system based on the stacking ensemble learning.
- *Neural collaborative filtering (NCF)* [27]: this work developed a recommender framework that uses the multi-layer perceptron to exploit the user-item interaction.
- *Variational Autoencoders for Collaborative Filtering* (MultiVAE) [15]: This approach investigates the collaborative information in a multinomial distribution to recommend items on the long tail.
- *Node2Vec embedding:* We propose a variant of MIGAN architecture, which deploys Node2Vec embedding representation instead of BGNN.

**Figure 4:**
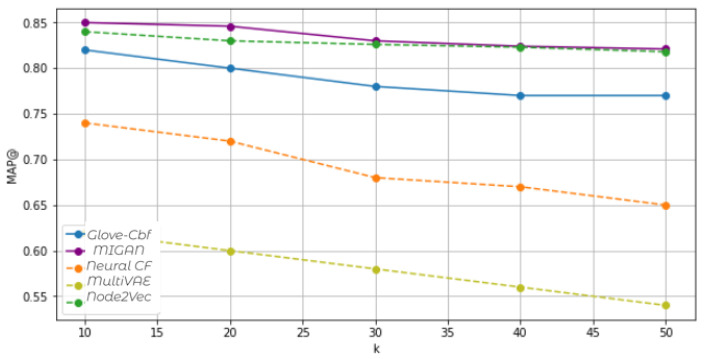
Performance results of Top-K recommended lists, according to MAP. The ranking position K ranges from 1 to 50.

Table 4 reports the performance of the various approaches under investigation using the MovieLens dataset. According to mean average precision, MIGAN outperforms the baselines. Indeed, it models the higher-order feature interactions. Figure 4 shows the MAP@k evaluation versus k-top items. As the MAP measure indicates the fraction of relevant articles in the top *k* suggestions averaged over all users, MIGAN creates a tailored recommendation. To put it differently, MIGAN outperforms the other models in recalling relevant items for the user. It retains the user–item interaction and generates a user-specific task recommendation. Furthermore, both recommender-based BGNN and the recommender-based Node2Vec are quite competitive. For example, MIGAN and Node2Vec achieve MAP@10 = 0.85, and MAP@10 = 0.84, respectively, outperforming the other baselines. The training loss equals 0.23 with dimensions d = 70 and 0.32 with d = 50 for BGGN and Node2vec, respectively. Node2Vec is a random walk-based node embedding method producing high memory consumption for large graphs. In contrast, the cascaded training used in BGGN does not involve loading the entire graph into memory. Consequently, it reduces the memory cost and training time. The MultiVAE recommender scores poorly. Indeed, it does not use a enough rich representation of data semantic. The neural collaborative filtering approach (Neural CF) shows a good score with k = 10, MAP@10 = 0.74 due to the appropriate representation of the interaction between user and item.

**Figure 5:**
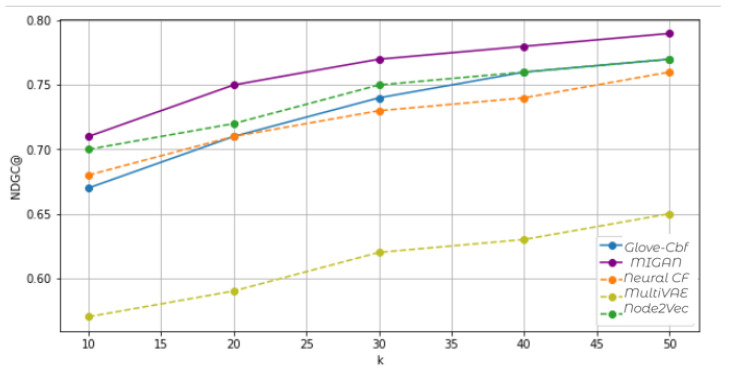
Performance results of Top-K recommended lists according to NDGC. The ranking position K ranges from 1 to 50.

Figure 5 shows that MIGAN exhibit a high NDCG score on MovieLens. Its Top-k recommendation list is quite similar to the ground-truth list. MIGAN boosts the modeling of user–item interaction. Indeed, the more interested the users are in an item, the more likely users with similar preferences to recommend it. Note that the Node2Vec also presents good NDCG scores. The graph recommender effectively obtains the user’s overall interest built by the neighborhood representation.

## 5. Conclusions

We propose and investigate a graph recommender where each user’s recommended content is accurate and personalized. MIGAN is a collaborative filtering (CF) system. A neural graph represents the item and users. It computes a representation of nodes by combining their neighborhoods’ vectors according to their mutual influence interaction by utilizing a co-attention mechanism with trainable attention weights. The attention weights are adjustable parameters computed by aggregating neighbor vectors. This neural graph architecture predicts the ratings assigned by the users to the items.

We perform a comparative evaluation of several configurations for the RS using the well-known dataset MovieLens. We use two metrics to quantify its accuracy: the mean average precision (MAP) and the normalized discounted cumulative gain (NDCG). The first is about the accuracy of the recommendation. The second is about the ranking of the recommended items. Comparing MIGAN performance with some baselines shows that it outperforms all its alternatives in MAP and NDGC scores. However, it would be very interesting to focus on a specific domain for recommendation tasks, such as taking the knowledge graph to distill attribute-based collaborative signals and compare MIGAN performance with knowledge graph attention network models. Thus, future work will investigate this framework for collaborative knowledge graphs involving contextual and semantic data. Future work will investigate this framework for other recommendation tasks involving contextual data.

## Figures and Tables

**Table 1:** Notation and descriptions.

Symbols	Definitions and Descriptions
$r_{ui}$	User *u*’s rating for item *i*
$p_{u}$	The user *u*’s embedding.
$q_{i}$	The item *i*’s embedding.
*g*	Long-Short-Term-Memory function
$x_{u}$	The user embedding layer followed by LSTM layer
$x_{i}$	The item embedding layer followed by LSTM layer
*h*	The Multi-Layer-Perception application
$lstm_{u}$	The user LSTM layer following by MLP
$lstm_{i}$	The item LSTM layer following by MLP
$\alpha_{u}^{*}$	Attention network function for user *u*
$\alpha_{i}^{*}$	Attention network function for item *i*
$\alpha_{u}$	The last attention weights for user *u*
$\alpha_{i}$	The last attention weights for item *i*
$C_{ui}$	User-item space
$C_{t}$	Text space
⊕	The concatenation operator
$r_{ui}^{{}^{\prime }}$	User *u*’s rating expected value for item *i*
W,b	The weight and bias in neural network
U,I	Nodes of bipartite graph
$X_{u},X_{i}$	lists of Features
$B_{u},B_{i}$	adjacency matrix

**Table 2:** MovieLens 1M description.

*# Users*	6040
*# Movies*	3883
*# Ratings*	1000209
*Sparsity %*	95.5%
*Item*	Genomic Tags
*User*	Demographics

**Table 3:** The best scoring MIGAN variant.

	Mean Average Precision			Normalized DCG		
**Variant**	MAP@10	MAP@30	MAP@50	NDCG@10	NDCG@30	NDCG@50
**Variant 1**	**0.85**	**0.83**	**0.81**	**0.71**	**0.78**	**0.79**
**Variant 2**	0.82	0.78	0.76	0.65	0.72	0.76
**Variant 3**	0.80	0.77	0.76	0.66	0.73	0.76
**Variant 4**	0.79	0.74	0.773	0.62	0.71	0.73
**Variant 5**	0.79	0.73	0.70	0.60	0.71	0.72
**Variant 6**	0.77	0.76	0.73	0.63	0.73	0.75

**Table 4:** Recommendation performance (%) of compared approaches conducted on MovieLens 1M dataset. We generate Top 10, 30, and 50 items for each user. The best score of MAP@k and NDCG@k are highlighted with a bold font.

	Mean Average Precision			Normalized DCG		
***Rec sys***	MAP@10	MAP@30	MAP@50	NDCG@10	NDCG@30	NDCG@50
**Glove-Cbf**	0.82	0.78	0.77	0.67	0.74	0.77
**Node2Vec**	0.84	0.82	0.81	0.55	0.65	0.69
**MultiVAE**	0.62	0.58	0.54	0.57	0.62	0.65
**Neural CF**	0.74	0.68	0.65	0.68	0.73	0.76
**MIGAN**	**0.85**	**0.83**	**0.81**	**0.71**	**0.78**	**0.79**

## Data Availability

The links to publicly datasets: https://dl.acm.org/doi/10.1145/2827872 (accessed on 1 March 2022).

## Abbreviations

The following abbreviations are used in this manuscript:

RS	Recommender Systems
MIGAN	Mutual-Interaction Graph Attention Network
BGNN	Bipartite Graph Neural Networks
IDMP	Inter-Domain Message Passing
IDA	Intra-Domain Alignment
LSTM	Long-Short-Term-Memory
GRU	Gate Recurrent Unit
MAP	Mean Average Precision
NDCG	Normalized Discounted Cumulative Gain
Neural-CF	Neural Collaborative Filtering
Glove-Cbf	Glove Content-based filtering recommender
MultiVAE	Variational Autoencoders recommender
